# ﻿Long-term stability in the winter diet of the Japanese serow (Artiodactyla, Caprinae)

**DOI:** 10.3897/zookeys.1122.76486

**Published:** 2022-09-20

**Authors:** Mitsuko Hiruma, Kahoko Tochigi, Ryosuke Kishimoto, Misako Kuroe, Bruna Elisa Trentin, Shinsuke Koike

**Affiliations:** 1 Graduate School of Agriculture, Tokyo University of Agriculture and Technology, 3-5-8 Saiwai-Cho, Fuchu, Tokyo 183-8509, Japan; 2 Institute of Mountain Science, Shinshu University, 8304 Minami-minowa, Kamiina-gun, Nagano 399-4511, Japan; 3 Nagano Environmental Conservation Research Institute, 2054-120 Kitagou, Nagano 381-0075, Japan; 4 Department of Ecology, UNESP São Paulo State University, Botucatu, São Paulo 18610-034, Brazil; 5 Institute of Agriculture, Tokyo University of Agriculture and Technology, 3-5-8 Saiwai-Cho, Fuchu, Tokyo 183-8509, Japan

**Keywords:** browser, *Capricorniscrispus*, *Cervusnippon*, population dynamics, sika deer, ungulate

## Abstract

The winter diets of northern ungulates are sensitive to changes in environmental conditions and ungulate population densities. We hypothesized that the winter diets of smaller browser ungulates might not readily change in response to fluctuating environmental conditions. We analyzed long-term trends in the winter diet of the Japanese serow (*Capricorniscrispus*) by analyzing rumen contents of 532 individuals over a span of 16 years among five populations along with changes in the population densities of sika deer (*Cervusnippon*) in Nagano Prefecture, central Japan. The winter diet composition of the serow was stable over the long term despite the increase in deer population density. The little-flexible nature of the serow diet may explain the long-term stability in the winter diets.

## ﻿Introduction

The winter diets of northern ungulates are sensitive to changes in environmental conditions, and the compositions of their diets undergo change to adapt to the nutritional restrictions of winter (e.g., Matinka 1968; Coblentz 1970; Morrison et al. 2002). Thus, nutrition is a critical link between environmental and population density in northern populations of free-ranging ungulates (Skogland 1985; Garroway and Broders 2005). Nevertheless, research on the winter food habits of northern free-ranging ungulates has mostly been limited to short-term studies.

Previous long-term studies on the winter diet of white-tailed deer (*Odocoileusvirginianus*) have shown that changes in vegetation alongside changing population densities and increasing snow depth can influence the winter diet (McCullough 1985; Tremblay et al. 2005; DelGiudice et al. 2012). Annual variations in the winter diet affect the deer’s nutritional intake, body condition, and population dynamics (Delgiudice et al. 2001). For sika deer (*Cervusnippon*), population density, snow depth, and habitat quality (e.g., vegetation structure) are critical factors that influence the composition of the winter diet (Seto et al. 2015). According to these studies, both grazers (northern sika deer) and browsers (white-tailed deer) can alter their winter diets according to changes in environmental conditions and population density (McCullough 1985; Tremblay et al. 2005; DelGiudice et al. 2012; Seto et al. 2015). White-tailed deer are medium-sized (50–130 kg) browsers that live in various habitats, such as forests and grasslands. They are nonterritorial, gather in herds, consume grasses, and migrate to areas that are rich in food resources (Smith 1991; VerCauteren and Hygnstrom 1998). However, smaller browsers tend to be solitary, territorial, inhabit forests, generally possess a higher-quality diet, and are highly selective in terms of food items and foraging areas (Jarman 1974; Hofmann 1989). We hypothesized that the winter diet of smaller and forest-dwelling browsers might not readily change in response to fluctuating environmental conditions.

The Japanese serow (*Capricorniscrispus*) is an endemic ungulate species in Japan. The Japanese serow was designated as a Special National Treasure in 1955; hunting this animal is illegal. In the early 20^th^ century, the number of serows decreased dramatically due to poaching, but their populations gradually recovered after protective legislation was passed after World War II (Tokida 2019). In the 1970s, their expanded distribution and increased population sparked conflicts with the forestry industry because serows eat the twigs and leaves of young trees in conifer plantations in the winter. Consequently, partial and facultative culling has been permitted since 1979 in damaged forest areas, and since 1990 in some damaged agricultural regions. Serows are small (30–50 kg) members of the family Bovidae and mainly inhabit forests. They are solitary, monogamous, and territorial. The habitat areas of male and female serows overlap; however, individual territories that are scent-marked with parotid gland secretions do not overlap with those of the same sex (Ochiai 2009). Serows are browsers that selectively feed on high-quality plant material; they mainly subsist on the leaves of deciduous broad-leaved trees and forbs from spring to fall and deciduous broad-leaved winter buds in the winter when food availability is low (Ochiai 1999). Depending on their habitat, they may also feed on evergreen conifers, evergreen shrubs, grasses, and sedges (Ochiai 1999; Jiang et al. 2008; Kobayashi and Takatsuki 2012; Asakura et al. 2014; Yamashiro et al. 2019). The leaves of conifers and broad-leaved evergreens can also serve as significant food resources for the serow (Jiang et al. 2008).

We examined the changes in serow food habits by analyzing long-term (2000–2015) trends in winter food habits in central Japan along with changes in the environmental conditions. Particularly, we focused on the population density of sika deer as an environmental condition. The serow is a browser that mainly feeds on woody plants and is considered to have a narrow food habit, whereas the sika deer is considered an “intermediate feeder” among cervid species with a very flexible food habit (Hofmann 1985). Thus, a previous study indicated that there is potential competition for food sources between serows and sika deer, and serows might not switch to alternative foods due to their narrow food habits if the number of deer populations becomes overabundant (Tokida 2019). Moreover, the study pointed out that this phenomenon may decrease the density of serows (Tokida 2019). However, it has not been definitively confirmed that the food habits of serows remain unchanged as the density of sika deer increases. We hypothesized that the winter food habits of serows do not change as the sika deer increases, because the serow is a small-sized, solitary, territorial, and forest-dwelling browser that is characterized by highly selective foraging habits. We examined the annual variation in winter diets of serows in Nagano Prefecture, central Japan, as well as the potential key variables that influence serow inhabitation.

## ﻿Methods

### ﻿Study area and population status of serow

**Figure 1.**
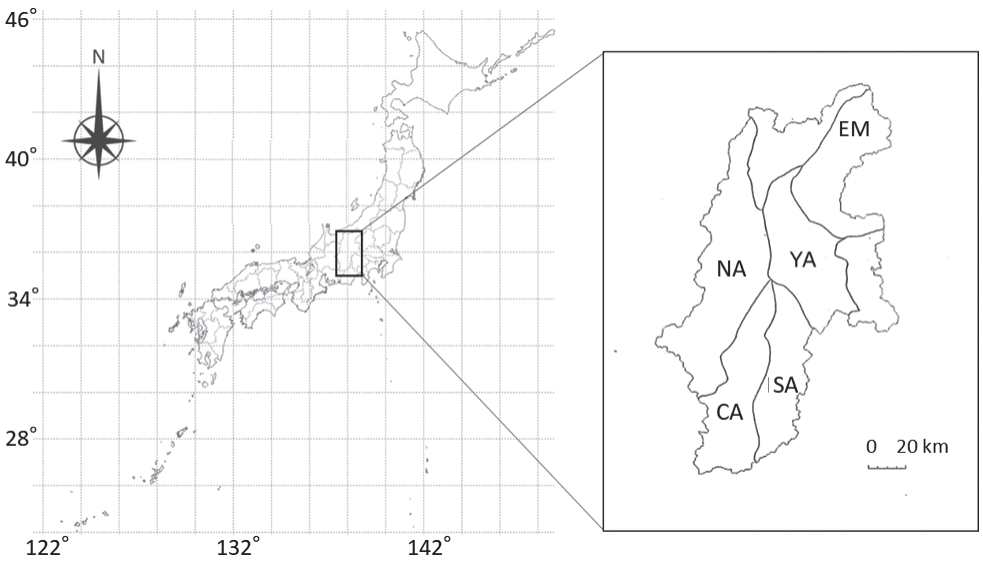
Location of Nagano Prefecture and location of the five serow populations analyzed: Echigo–Nikko–Mikuni (EM), South Alps (SA), North Alps (NA), Central Alps (CA), and Yatsugatake (YA).

Nagano Prefecture, Japan (35°11'–37°01'N, 137°19'–138°44'E) (Fig. 1), covers a wide latitudinal area, with a climate that differs markedly from north to south. The annual mean temperature, precipitation, and snow accumulation for the years 2005–2015 were 12.2 °C, 81.3 mm, and 14.4 cm depth, respectively, in the northern region (36°39'N, 138°11'E); 11.4 °C, 107.7 mm, and 7.7 cm depth, respectively, in the central region (36°02'N, 138°06'E); and 13.0 °C, 138.6 mm, and 5.4 cm depth, respectively, in the southern region (35°31'N, 137°49'E) (Japan Meteorological Agency 2019). No statistically significant trend was highlighted in long-term changes in the annual greatest snow depth in Nagano Prefecture (Shinshu Monitoring Network for Climate Change 2018).

The estimated total serow population in Nagano Prefecture was 9340 ± 1630 in 2000 but has hovered around 7738 ± 6420 in 2018 using the block-count method (Nagano Prefecture 2020). There has been little change in the distribution area of serows over the past 20 years (Nagano Prefecture 2020). There are seven local serow populations in Nagano Prefecture: Echigo–Nikko–Mikuni (EN), South Alps (SA), North Alps (NA), Central Alps (CA), Yatsugatake (YA), Kanto Mountains, and Northern Nagano (Nagano Prefecture 2020). The Kanto Mountains and Northern Nagano populations were excluded from this study because the sample numbers of the populations were too small for data analysis. The results of an official national survey using the block-count method (Maruyama and Furubayashi 1983) after fall defoliation revealed gradual long-term declines from 2000 to 2014 in all populations. The densities (serows / km^2^ ± SD) of the populations in 2000, 2004, and 2014, are shown in Table 1 (Nagano Prefecture 2000, 2020).

**Table 1.** Estimated densities (serows / km^2^ ± SD) of the serow populations in 2000, 2004, and 2014 (Nagano Prefecture 2000, 2020).

	EM	SA	YA	NA	CA
2000	2.48 ± 3.43	0.57 ± 0.83	0.47 ± 1.04	1.70 ± 1.99	4.16 ± 3.23
2004	0.51	0.33 ± 0.67	0.46 ± 0.73	0.51 ± 0.61	2.40 ± 2.37
2014	0.83 ± 1.11	0.25 ± 0.70	0.28 ±0.49	0.59 ± 1.00	2.22 ± 2.84

Echigo–Nikko–Mikuni (EM), South Alps (SA), Yatsugatake (YA), North Alps (NA), and Central Alps (CA). Estimated number and density (number/km^2^) based on the block count method.

### ﻿Serow rumen samples

**Figure 2.**
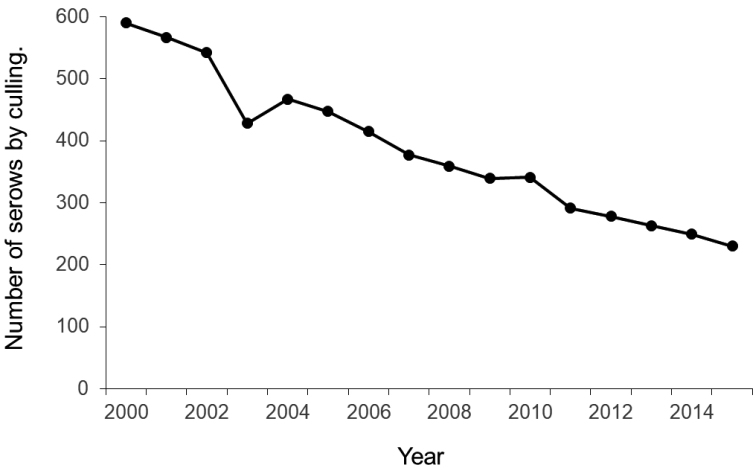
Number of serows through culling from 2000 to 2014 (Nagano Prefecture 2020).

Since 2000, approximately 10% of the rumen contents has been collected randomly from each culled serow for monitoring purposes (Fig. 2). We used rumen content samples from serows culled in forest areas from 2000 to 2015 for analysis. The sample numbers for each population are as follows: EN, 99; SA, 71; NA, 148; CA, 160; and YA, 54. In total, we analyzed 532 samples.

We analyzed the reported frequencies as percentages of each food group using the point-frame method (Stewart 1967), which estimates the percentage composition according to the surface area and is easier to conduct than weight-based methods. We randomly selected subsamples from each sample, spread them over 5 mm grids, and then identified the plant fragments touching each intersection point along a transect. We evaluated each subsample at 400 points or more per transect. Since we could not identify most plant fragments to the species level, we categorized them into six food groups: i.e., leaves of deciduous broad-leaved trees, leaves of broad-leaved evergreens trees, leaves of planted conifers, leaves of natural conifers, leaves of graminoids, and other materials. The other food materials include woody fibers, woody buds, fruits, ferns, and unknown items. Planted conifers refer to *Cryptomeriajaponica* and *Chamaecyparisobtusa*, whereas natural conifers comprise other conifer species.

### ﻿Deer density

For data regarding sika deer population density, we referred to the official surveys conducted by Nagano Prefecture (Table 2) (Nagano Prefecture 2016). These surveys grouped deer into seven populations that nearly overlap with the serow populations; therefore, we used the deer density for the deer population (hereafter, deer density) that corresponded to the target serow population. We used the values for the years that the survey was conducted (1999, 2010, and 2015) to estimate a regression model in which the value for the no-survey years was the dependent variable and the value for the recorded year was the explanatory variable. We then used the value estimated by the said regression model (theoretical value on the regression line) as the assigned value.

**Table 2.** Estimated number and density (median values) of sika deer in each area of Nagano Prefecture in 1999, 2010, and 2015 (Nagano Prefecture 2016).

	YA	SA	Other
1999	8,657 (6.2)	18,858 (11.2)	–
2010	48,527 (20.7)	33,787 (11.4)	8,644 (3.9)
2015	128,598 (51.4)	30,812 (12.7)	19,795 (5.2)

Yatsugatake (YA) and South Alps (SA) Estimated number and density (number/km^2^) based on the block count and fecal pellet methods.

**Figure 3.**
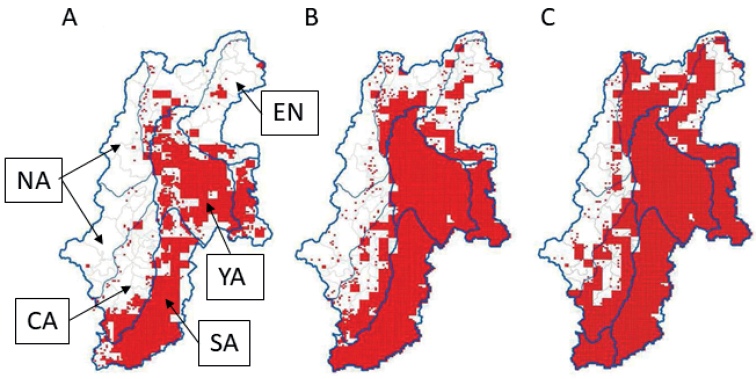
Distribution of sika deer in Nagano Prefecture in 2003 (**A**), 2010 (**B**), and 2015 (**C**). Red area: the distribution meshes (1 km^2^: 1 km × 1 km) of the existence or non-existence of sika deer based on a questionnaire completed by local people and hunting statistic data (Nagano Prefecture 2016).

The results of an official survey using the block count and fecal pellet count methods in the fall of each year (1999–2015) revealed that there was a gradual long-term increase in the deer population in YA, whereas the densities of the SA population remained stable, and the densities of the NA, CA, and EN populations remained low. Additionally, most areas were not inhabited by deer until the 2000s (Fig. 3) (Nagano Prefecture 2016).

### ﻿Statistical analysis

For analysis, years potentially have both a direct effect through the decrease of food availability and an indirect effect thorough deer density on the winter diet of serows. We employed Bayesian regression models with paths to verify the existence or non-existence of this relationship.

First, we converted the food item frequency of each food group to proportions and modeled them with Dirichlet regression to account for the composition data. Since the number of rumen content counts at cross-sectional points is equal to the discrete level, and the total number of counts for each subsample is summed up to a fixed number, the probability of the occurrence of one group is influenced by increases or decreases in other groups (i.e., multivariate analysis). A disadvantage of this transformation is that the information from the data is changed or lost when skewing the data structure. In contrast, the Dirichlet distribution is appropriate for count-based data summed up to a fixed value.

To build a model for estimating the factors influencing diets of serows, we set the proportion of the six food groups as the response variables. In food groups, we set leaves of deciduous broad-leaved trees as the reference category. We defined year (the years 2000–2015 were converted to years 1–16) and deer density as the explanatory variables. Food composition is possibly different among serow populations due to differences in vegetation and food availability. Therefore, we included the serow population as a random effect. We assumed that response variables follow a Dirichlet distribution.

To build a model for estimating annual changes in deer density, we set the deer density as a response variable and year as an explanatory variable. Density may differ among deer populations due to differences in hunting pressure and food availability. Therefore, we included the serow population as a random effect. We assumed that response variables follow a normal distribution.

To evaluate whether there are significant paths among serow dietary composition, year, and deer density, we checked whether 95% credible intervals (CIs) for estimate values of each variable were greater than zero. We standardized the estimated unstandardized coefficients to compare the impact of factors in which the unit and size differed. We used a Bayesian approach, implemented using the BRMS (Bayesian Regression Model Stan) package (Bürkner 2018) in R (R Core Team 2021). We used the BRMS default priors for each regression. We set the number of iterations to 30,000 and discarded the first 20,000 steps as burn-in. Then, we thinned the remaining 10,000 steps every 50 steps to give 1000 samplings per chain. Subsequently, we ran four chains with randomly selected initial values for each model. Finally, we evaluated whether MCMC converged well from visual assessment using the Gelman–Rubin diagnostic (1.00–1.08) (Gelman et al. 2003).

## ﻿Results

**Figure 4.**
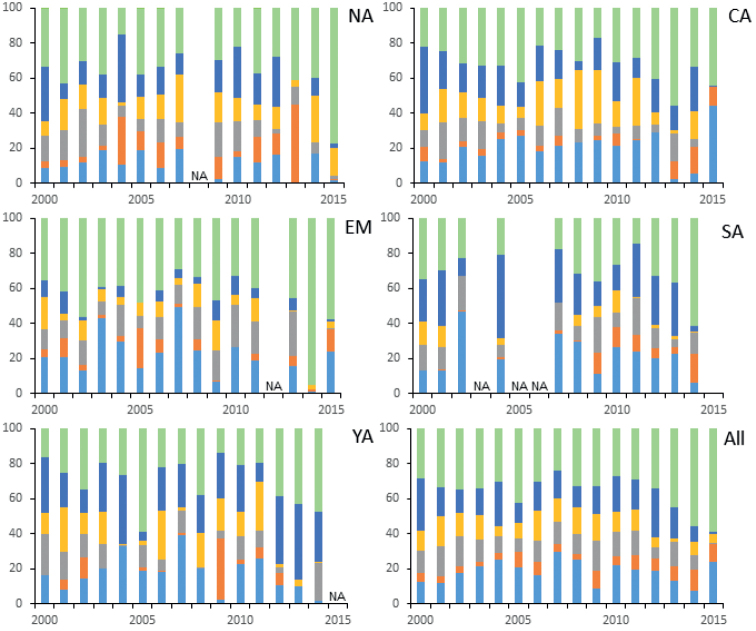
Percent composition of the six major food groups in Japanese serow rumen samples of the six populations in Nagano Prefecture, pooled across the populations from 2000 to 2015. The leaves of different plants are depicted as follows. Aqua: deciduous broad-leaved trees, orange: broad-leaved evergreens trees, gray: planted conifers, yellow: natural conifers, blue: graminoids, yellow: other plant foods. The populations are North Alps (NA), Central Alps (CA), Echigo–Nikko–Mikuni (EN), South Alps (SA), Yatsugatake (YA), and pooled across all populations (All).

**Figure 5.**
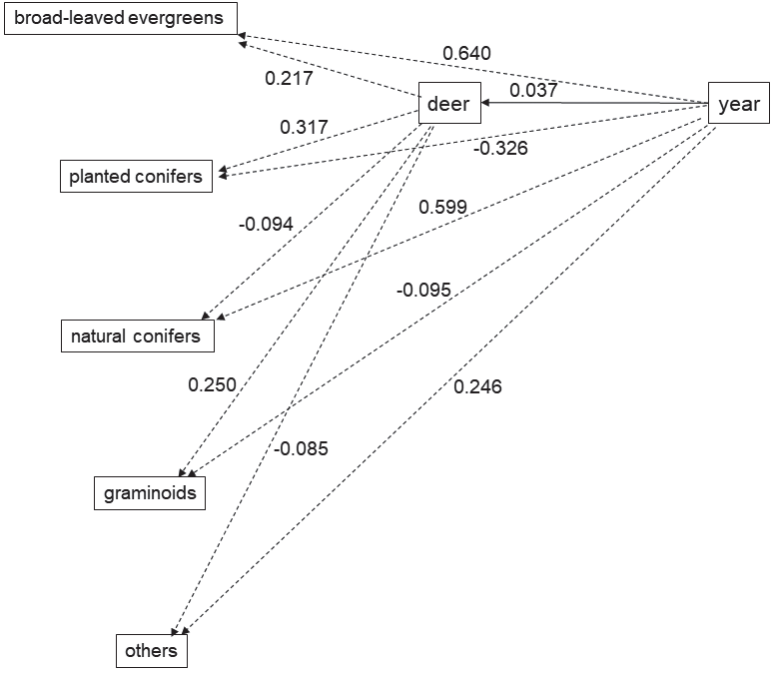
Directed acyclic graph depicting the year and deer density that are not important in the path analysis of serow food habits. Solid lines: 95% credible intervals (CIs) whose path coefficients do not overlap with zero (see Table 2). Dash lines: coefficients whose 95% CIs overlap with zero. The larger the absolute value of the coefficient, the stronger the effect.

According to the model analysis results, the 95% CIs of coefficients of year and deer density on all food groups had zero overlap (Table 3). In other words, the composition of food in the serow rumens did not change linearly over the years and was not affected by deer density (Fig. 4). The effect of year on deer density was significant (unstandardized mean = 0.058; 95% CI: 0.039, 0.075, Table 3). Therefore, only the direct path of year to deer density was significant. These results are due to the fact that we had to estimate the no-survey years of deer density by regression imputation. The direct path of the year to serow diets and the indirect path of the year through deer density to serow food composition were not significant (Fig. 5).

**Table 3.** Model coefficients for six the food plat groups of the serow rumen modeled with Dirichlet distribution for deer density and with normal distribution. Leaves of deciduous broad-leaved trees were set as the reference category. We presented estimates in 95% credible intervals (CIs) for each model parameters. All response variables had a random effect on population. Estimated coefficients were standardized to have zero mean with a standard deviation 1.

Response variables	Parameter	Estimate	SE	Lower CI	Upper CI	Standardized estimate
**Fixed effect**						
Leaves of broad-leaved evergreen trees	Intercept	−1.032	0.163	−1.360	−0.737	0.000
	Year	0.019	0.014	−0.008	0.046	0.640
	Deer density	−0.004	0.010	−0.024	0.017	−0.217
Leaves of planted conifers	Intercept	−0.342	0.135	−0.628	−0.085	0.000
	Year	−0.013	0.014	−0.042	0.013	−0.326
	Deer density	0.008	0.010	−0.011	0.028	0.317
Leaves of natural conifers trees	Intercept	−0.734	0.211	−1.139	−0.320	0.000
	Year	0.024	0.014	−0.003	0.051	0.559
	Deer density	−0.003	0.010	−0.021	0.017	−0.094
Leaves of graminoids	Intercept	−0.312	0.243	−0.792	0.210	0.000
	Year	−0.005	0.014	−0.031	0.022	−0.095
	Deer density	0.008	0.010	−0.012	0.027	0.250
Other food material	Intercept	0.491	0.166	0.139	0.789	0.000
	Year	0.013	0.012	−0.011	0.038	0.246
	Deer density	−0.003	0.010	−0.022	0.017	−0.085
Deer density	Intercept	1.033	0.888	−0.964	2.780	0.000
	Year	0.058	0.009	0.039	0.075	0.037
**Random effect**						
SD (Leaves of broad-leaved evergreen trees)	Intercept	0.187	0.189	0.011	0.705	
SD (Leaves of planted conifers trees)	Intercept	0.129	0.114	0.005	0.407	
SD (Leaves of natural conifers)	Intercept	0.317	0.208	0.083	0.873	
SD (Leaves of graminoids)	Intercept	0.421	0.277	0.134	1.200	
SD (Other food material)	Intercept	0.219	0.195	0.015	0.760	
SD (Deer density)	Intercept	1.864	0.898	0.800	4.204	

## ﻿Discussion

The serows’ winter diet did not readily change in response to increasing deer density. This may be a result of their ecological characteristics, such as small size, solitary nature, and territoriality, which characterized highly selective foraging habits. Therefore, the results support our hypothesis.

It is well known that when deer density rises, vegetation, particularly forest floor vegetation, declines or disappears (Takatsuki 2009). In Nagano Prefecture, there is evidence that the forest ground vegetation is decreasing due to the increasing deer population (Nagano Prefecture 2016). Previous studies on the degree of dietary overlap between serows and sika deer revealed the existence of a dietary partition between the two species (Kobayashi and Takatsuki 2012). However, due to the vast range and flexibility of feeding habits in deer, they expanded their diet to include coniferous tree needles and twigs and bark of deciduous trees only during severe winters that is limited food resources season (Koganezawa 1999). As a result, their diet overlapped with that of the serows during these winters, possibly resulting in a rapid depletion of the serows’ restricted food supply due to the expanded consumption of food items of sika deer. In accordance with the findings from the previous studies, the results of this study suggest the following two possibilities by deer density increase for serows’ response occurs separately or simultaneously.

First, spatial partitioning between sika deer and serows may progress. Although there are some regions where the diets of both species overlap, distinctions in habitat use between the two species are also known (Yamashiro et al. 2019). Specifically, there are differences in habitat use between the two species; serows utilized steep rocky slopes, whereas sika deer appeared more frequently in grasslands (Yamashiro et al. 2019; Takada et al. 2020). Due to this spatial partitioning, increase of sika deer population might not have affected the winter diet of serow populations. Second, as serows strictly defend their home ranges as intrasexual territories and are very sedentary (Kishimoto and Kawamichi 1996), it is assumed that serows may respond to decrease food amount caused by an increase deer density by expanding their territorial range. This is supported by the positive correlation between food amount and serows density and negative correlation between food amount and serows’ territorial range size (Sone et al. 1999; Ochiai et al. 2010). Therefore, if food availability deteriorates to the point where the serows cannot maintain territory, serows may need to move into the more inhospitable region. The occurrence of these two events may have caused a long-term decline in serow population.

In this study, we could not clearly evaluate the relationship between the increase in deer and the decrease in serows because we did not investigate changes in the vegetation structure and winter food habits of deer and serows’ behavior. However, decrease in serow food supply as a result of increased sika deer density could have resulted in a spatial partitioning between sika deer and serow or decrease in serow density.
